# Nonenteric Pathogens in Urinary Tract Infections: Epidemiology and Resistance Patterns in Albania

**DOI:** 10.1155/ghe3/9944598

**Published:** 2025-04-17

**Authors:** Silvi Bozo, Irida Ikonomi Hoxha, Eftiola Pojani

**Affiliations:** ^1^Department of Chemical-Pharmaceutical and Biomolecular Technologies, Faculty of Pharmacy, Catholic University “Our Lady of Good Counsel”, Tirana, Albania; ^2^Department of Medical Technical Sciences, Faculty of Professional Studies, “Aleksandër Moisiu” University, Durrës, Albania

**Keywords:** Albania, antibiotic resistance, MDR, nonenteric pathogens, UTIs

## Abstract

**Introduction:** Antibiotic resistance is a growing global health crisis that complicates the treatment of urinary tract infections (UTIs). While Enterobacterales are primary UTI pathogens, nonenteric pathogens such as *Pseudomonas aeruginosa*, *Burkholderia cepacia*, and *Enterococcus* spp. are increasingly recognized, posing challenges due to their complex resistance mechanisms. This study aims to investigate the prevalence, resistance patterns, and multidrug resistance (MDR) of nonenteric pathogens in community-acquired UTIs in Albania.

**Materials and Methods:** The study was conducted in an outpatient clinic from September 2023 to September 2024, involving adults (≥ 18 years) and excluding individuals with recent antibiotic use or pregnancy. Urine samples were processed using blood and MacConkey Agar, followed by bacterial identification and susceptibility testing with the VITEK 2 system. A total of 11 antibiotics belonging to *β*-lactams, fluoroquinolones, glycylcyclines, oxazolidinones, lipopeptides, glycopeptides, and tetracyclines were tested. Statistical analysis was performed using SPSS, with significance set at *p* < 0.05.

**Results:** A total of 550 urine cultures were analyzed, of which 372 (67.6%) were positive for bacterial growth. Among these, 27.7% were identified as nonenteric pathogens, with a higher occurrence in females (66%) and young adults (18–39 years) (60.2%). *Enterococcus faecalis* was the most common Gram-positive pathogen (15.2% of the positive samples), while *P. aeruginosa* was the most frequent Gram-negative pathogen (9.1%). *P. aeruginosa* showed significant resistance to tigecycline (91.2%) and levofloxacin (38.2%), with no resistance to meropenem. *E. faecalis* showed high resistance to vancomycin (53.6%) and teicoplanin (46.4%), while *S. saprophyticus* showed moderate resistance. MDR prevalence was highest in *P. aeruginosa* (26.5%).

**Conclusion:** This study highlights the high prevalence of community-acquired UTIs in Albania, particularly among females, and concerning MDR rates. To address these challenges, it is crucial to implement standardized treatment protocols, improve antibiotic stewardship, and promote research to track resistance patterns, ultimately enhancing patient care and combating antibiotic resistance.

## 1. Introduction

Antibiotic resistance has emerged as one of the most pressing global health challenges, significantly complicating the management of urinary tract infections (UTIs). UTIs represent a substantial healthcare burden worldwide, impacting millions annually and often leading to increased hospitalizations, healthcare costs, and adverse outcomes [1, 2]. Although Enterobacterales, such as *Escherichia coli (E. coli)* and *Klebsiella pneumoniae (K. pneumoniae)*, are the primary causative agents of UTIs, a growing body of evidence highlights the significant role of nonenteric pathogens in these infections [3, 4]. Nonenteric pathogens are microorganisms that do not primarily reside in the intestinal tract and include *Pseudomonas aeruginosa (P. aeruginosa)*, *Burkholderia cepacia (B. cepacia)*, and certain Gram-positive bacteria such as *Enterococcus* spp. These pathogens not only contribute to the complexity of UTIs but also present unique challenges due to their inherent and acquired resistance mechanisms [5].

According to the Global Research Agenda for Antimicrobial Resistance in Human Health, Policy Brief 2023, one of the World Health Organization's (WHO's) priorities is to investigate the prevalence of community-acquired UTIs (CA-UTIs) caused by antibiotic-resistant WHO priority pathogens such as *P. aeruginosa*, with data disaggregated by demographic characteristics [6].

Multidrug-resistant (MDR) bacterial infections present a major challenge to healthcare systems worldwide, particularly in the case of wound infections. These infections contribute to longer hospital stays, higher rates of morbidity and mortality, and increased healthcare costs [7]. Similarly, the increasing prevalence of resistant nonenteric bacteria in UTIs exacerbates these challenges. These pathogens often exhibit intrinsic resistance mechanisms such as efflux pumps and *β*-lactamase production, allowing them to withstand multiple classes of antibiotics, including beta-lactams, fluoroquinolones, and even last-resort treatments like carbapenems [8]. Such resistance patterns can lead to prolonged infections, recurrent UTI episodes, and higher risks of complications, particularly in regions with limited access to novel antimicrobials. Furthermore, the economic burden associated with managing these infections is significant, as healthcare systems struggle with the costs of prolonged hospitalizations and the use of more expensive antibiotics [9]. The financial implications are aggravated by the need for alternative therapies, which may not be readily available in all healthcare settings, particularly in developing countries [10].

The epidemiological landscape of UTI pathogens in Albania is characterized by a complex interplay of factors, including antibiotic prescribing practices, patient demographics, and healthcare infrastructure. Prior antibiotic exposure is a significant risk factor for acquiring resistant organisms, underscoring the importance of rational antibiotic use in both community and hospital settings [11]. Additionally, the lack of access to effective antimicrobials in certain healthcare facilities exacerbates the problem, limiting treatment options for patients with resistant infections [12].

CA-UTIs, defined as infections of the urinary tract that occur in the community or within less than 48 h of hospital admission, are among the most commonly diagnosed infections in community settings, following respiratory infections [13, 14]. Studies indicate that CA-UTIs account for a significant proportion of all UTIs diagnosed annually, highlighting their relevance in both clinical practice and public health [15].

In Albania, studies focused on the epidemiology and resistance profiles of UTIs are very scarce, and there is a complete absence of research specifically addressing nonenteric pathogens associated with these CA-UTIs. To the best of our knowledge, no guidelines or stewardship programs have been implemented in the country to effectively address antibiotic resistance. Understanding local prevalence and resistance trends is essential for developing effective treatment protocols and guiding empirical antibiotic selection, particularly in regions with limited data [16]. This knowledge supports the development of infection control policies and antimicrobial stewardship tailored to the Albanian healthcare setting. Addressing these gaps is crucial for evidence-based UTI management and reducing the public health impact of resistant nonenteric infections. Moreover, such data contribute to global antibiotic resistance monitoring, offering insights into underrepresented regions and guiding international treatment strategies.

This study aims to address the above urgent needs by evaluating the prevalence, resistance patterns, and MDR rates of nonenteric pathogens associated with CA-UTIs in Albania.

## 2. Materials and Methods

### 2.1. Study Design

This study was conducted in an outpatient urban healthcare clinic in Albania, with sample collection carried out from September 2023 to September 2024. Ethical approval was granted by the University “Aleksandër Moisiu” Ethical Council, Republic of Albania (Prot. Nr. 135/520 on 30/05/2024). Informed consent was obtained from all participants after they were briefed on the study's objectives, with full respect for privacy and confidentiality.

Patients self-referred to the clinic based on their personal evaluation of symptoms, with dysuria being the most frequent symptom prompting them to seek analysis.

Adults, aged 18 years and older, were eligible for inclusion in the study. Only one urine culture per patient was included to ensure that each sample represented a unique individual. Participants who had taken antibiotics within three days prior to sample collection and pregnant women were excluded. Age categories were defined as follows: young adults (18–39 years), adults (40–64 years), and elderly (≥ 65 years).

### 2.2. Sample Collection and Processing

All analyses were performed in an ISO-certified laboratory. Midstream urine samples were collected from eligible participants. Samples were immediately transported to the laboratory under controlled conditions and processed within 2 h of collection. For inoculation, a calibrated loop with a capacity of 10 μL (0.01 mL) was used for culture and analysis. Urine cultures were performed using blood agar for Gram-positive bacteria and MacConkey agar for Gram-negative bacteria. The parameters for culture included incubation conditions (aerobically at 37°C for 24 h), acceptable culture media, culture age, and inoculum turbidity. A bacterial count of 100,000 CFU/mL was considered positive for infection. In all cultures analyzed, a single bacterial species was identified, indicating that each positive urine sample was exclusively associated with one organism, and no instances of mixed infections were observed.

### 2.3. Bacterial Identification and Susceptibility Testing

Bacterial identification and susceptibility testing were performed using the VITEK 2 automated system (BioMérieux, France), following the manufacturer's guidelines. The antibiotics tested were selected based on the cards provided by the manufacturer for each bacterial species. The following antibiotics were tested for Gram-negative bacteria: ticarcillin/clavulanic acid, piperacillin, cefepime, meropenem, levofloxacin, and tigecycline, while Gram-positive bacteria were tested against levofloxacin, linezolid, daptomycin, teicoplanin, vancomycin, tetracycline, and tigecycline.

A sterile saline solution was used to suspend bacterial colonies from pure cultures, and subsequently, the turbidity was measured using a DensiChek turbidity meter (BioMérieux, France). Bacterial suspensions were injected into VITEK 2 identification cards, and the system performed kinetic analysis, reading each test every 15 min. Results were interpreted following the guidelines of the Clinical and Laboratory Standards Institute (CLSI) [17]. MDR strains were defined as microorganisms with nonsusceptibility to at least one antibiotic in three or more distinct antimicrobial categories [18].

### 2.4. Statistical Analysis

Data were analyzed using the Statistical Package for the Social Sciences (SPSS), Version 27.

Descriptive statistics were used to present demographic characteristics, stratified by bacterial type. The prevalence of bacterial species was expressed as frequencies relative to the total number of positive samples, while resistance patterns were expressed as frequencies within each bacterial type. The correlation between demographic factors, bacterial types, and resistance patterns was evaluated using chi-square or Fisher's exact test. A *p* value of < 0.05 was considered statistically significant.

## 3. Results

A total of 550 urine cultures were analyzed to evaluate the prevalence and characteristics of UTIs within the sampled population.

Out of these, 372 samples tested positive for CA-UTI, resulting in a prevalence rate of 67.6% (95% CI: 63.5–71.5). 103 isolates, representing 27.7% of the positive samples, were identified as nonenteric pathogens, including both Gram-positive and Gram-negative bacteria.  Table 1 presents the distribution of microorganisms according to age and gender. These microorganisms were predominantly isolated in females (66%) compared to males (34%). The age distribution showed that most cases (60.2%) occurred in younger individuals, aged 18–39 years, while the least affected group was the elderly, aged 65 years or older (14.6%).

Single-species isolates were most identified in females for all pathogens. Age and gender did not show a significant association with Gram-negative pathogens (age: *p*=1.544, gender: *p*=0.072). Similarly, no significant association was found between age, gender, and Gram-positive pathogens (age: *p*=1.464, gender: *p*=1.140).

Among the Gram-positive bacteria, *Enterococcus faecalis (E. faecalis)* was the most prevalent, representing 15.2% of the total positive samples, followed by *Staphylococcus saprophyticus (S. saprophyticus)* (3.2%). Among the Gram-negative bacteria, *P. aeruginosa* was the most frequently isolated organism (9.1%), while only 1 strain (0.3%) belonged to *B. cepacia*.

The antibiotic resistance patterns of four key pathogens were examined across various antibiotic classes, stratified by gender and age groups (Table 2). Among the pathogens analyzed, *P. aeruginosa* showed higher resistance patterns. Over 90% of strains (91.2%) were resistant to tigecycline, and 38.2% were resistant to levofloxacin. High resistance was also reported for ticarcillin/clavulanic acid and piperacillin (26.5%). Significant gender differences in resistance rates were observed for ticarcillin/clavulanic acid (*p*=0.012) and piperacillin (*p*=0.049), with females showing higher resistance prevalence. No resistance was detected for meropenem.

In contrast, the single strain of *B. cepacia* isolated in this study showed no resistance to any of the antibiotics tested.

Among the Gram-positive bacteria, *E. faecalis* showed the highest resistance rates to vancomycin (53.6%), teicoplanin (46.4%), and tetracycline (44.6%). Resistance to levofloxacin (14.3%) and linezolid (17.9%) was moderate. Only one strain (1.8%) was resistant to tigecycline, while all strains were sensitive to daptomycin. Statistically significant gender differences were found for levofloxacin (*p*=0.002), with a higher prevalence of resistance in females (67.9%) compared to males.

*S. saprophyticus* showed moderate resistance to the antibiotics tested, including teicoplanin (25.0%), levofloxacin (16.7%), linezolid (16.7%), vancomycin (16.7%), tigecycline (16.7%), and tetracycline (16.7%), with no resistance observed for daptomycin. No statistically significant correlations were found between resistance rates and age or gender for *S. saprophyticus*.

Finally, the prevalence of MDR strains was evaluated among the identified uropathogens. *P. aeruginosa* had the highest MDR rate at 26.5%, while *B. cepacia* showed no resistance (0%). *E. faecalis* and *S. saprophyticus* had a similar MDR prevalence rate of 16.1% and 16.7%, respectively.

## 4. Discussion

The urine culture analysis in this study identified a high prevalence of CA-UTIs at 67.6%, particularly among females (66%). This prevalence is notably higher than the reported global prevalence of 0.7% [19]. However, direct comparisons are challenging due to significant variations in epidemiological patterns and healthcare practices across countries. For instance, in the United States, CA-UTIs represent a substantial portion of all UTIs, with approximately 50% of women experiencing at least one UTI in their lifetime and an annual incidence of around 10 million cases [20]. In Europe, prevalence rates also vary widely; a nationwide German study reported that 60% of females had positive urine cultures, while the ECO.SENS study in Ireland observed a significant proportion of acute CA-UTIs among 4734 women aged 18–65 years, although specific prevalence rates were not detailed [21, 22].

The predominance of UTIs in females, as observed in this and other studies, is attributable to anatomical and physiological factors such as the shorter urethra and hormonal influences on the urinary tract microbiome [23, 24]. Moreover, the age distribution in this study, with 60.2% of cases occurring in individuals aged 18–39 years, is consistent with previous findings reporting the highest infection rate (52.4%) in women aged 25–34, a pattern linked to increased reproductive and sexual activity during this life stage [25].

Evidence consistently shows that a considerable percentage of pathogens identified in UTIs do not belong to Enterobacterales, highlighting the importance of recognizing the diversity of uropathogens in clinical practice [26–28]. The identification of nonenteric pathogens in 27.7% of the positive samples in our study confirms that even in Albania, these pathogens contribute to the emergence of UTIs. Moreover, the presence of Gram-positive bacteria, particularly *E. faecalis* (15.2% of the total positive samples), underscores the evolving nature of UTI pathogens. *Enterococcus* species are recognized as significant contributors to both hospital-acquired and CA-UTIs, with *E. faecalis* being the most prevalent among enterococci [29]. Among Gram-negative nonenteric bacteria, *P. aeruginosa* was the most frequently identified species (9.1%). This prevalence appears higher than reports from certain regions. For example, Hrbacek et al. documented a prevalence of 7.3% in Central Europe [30]. However, a ten-year surveillance study by Linhares et al. highlighted that while *P. aeruginosa* remains an uncommon cause of CA-UTIs (2.4%), there has been a significant increase in its incidence [31].

Based on the results presented earlier, this study further investigated resistance patterns among various pathogens. *P. aeruginosa* showed concerning resistance patterns, with more than 90% of strains resistant to tigecycline, over 38% resistant to levofloxacin, and more than 26% resistant to penicillins. The reduced susceptibility of this bacterium to tigecycline is potentially due to resistance associated with the nodulation division family of efflux pumps [32]. Countries, particularly in Southeastern Europe, have also reported high resistance rates in *P. aeruginosa*, especially to fluoroquinolones and other critical antibiotics, highlighting the pathogen's adaptability and resilience in clinical settings [33]. The resistance to fluoroquinolones observed in our study is particularly concerning, as empirical use for uncomplicated UTIs is typically recommended when resistance rates are below 20% [34]. On the other hand, the absence of resistance to meropenem in this study suggests that carbapenems may still be effective against *P. aeruginosa* in the Albanian context, in contrast to other countries with higher resistance patterns [5]. Furthermore, the statistically significant gender differences in resistance rates for penicillins, with females showing higher resistance prevalence, add complexity to the understanding of antibiotic resistance. As mentioned previously, this could be due to several biological and behavioral factors that may contribute to gender differences in resistance patterns. In our study, *P. aeruginosa* showed the highest MDR rate at 26.5%, which is significantly higher than the global rate of 13.4% reported in the European Centre for Disease Prevention and Control (ECDC) annual report [35]. This situation is particularly concerning as it limits the effectiveness of first-line empirical therapies, requiring the use of more potent and potentially toxic alternatives, which may be subject to limitations in availability in Albania, a situation previously reported in the country [12].

In contrast, the single strain of *B. cepacia* isolated in this study was sensitive to all the antibiotics tested. This finding is particularly interesting given that *B. cepacia* is recognized for its significant intrinsic resistance, placing it among the most highly antimicrobial-resistant organisms [36]. This suggests that the strain analyzed in our case may be atypical or that local factors influence its resistance profile. However, a single strain cannot account for these variations, requiring further investigation to confirm these local resistance patterns.

Among Gram-positive bacteria, *E. faecalis* showed high resistance rates, particularly to vancomycin (53.6%) and teicoplanin (46.4%), which is worrisome given the critical role of these antibiotics in treating enterococcal infections. Pontefract et al. suggested that linezolid could be an effective alternative for treating mild vancomycin-resistant *Enterococcus* UTIs [37]. While both studies highlight significant resistance to vancomycin, alternative treatment options, such as linezolid, offer potential solutions where traditional therapies may fail. However, the effectiveness of linezolid in our local context requires further investigation, given the reported resistance rate of 17.9%. Moreover, the moderate resistance to levofloxacin (14.3%) further contributes to existing concerns, as the European Association of Urology (EAU) guidelines recommend empirical use of quinolones only if the local resistance rate is below 10% [38]. The significant gender differences noted for levofloxacin resistance, with a higher prevalence in females (67.9%), further emphasize the need for gender-specific considerations in developing personalized treatment approaches, even for this strain.

*S. saprophyticus* showed moderate resistance to several antibiotics with a lower overall resistance compared to *E. faecalis*. The highest resistance rate was observed for teicoplanin (25.0%). This uropathogen is particularly common among young women, as seen in the group included in our study, further emphasizing its clinical relevance in this population [39]. Although teicoplanin is not typically a first-line treatment for CA-UTIs, it is often used for resistant Gram-positive infections, making its resistance a concerning issue [40]. This pattern suggests that *S. saprophyticus* may be evolving in response to antibiotic pressure, potentially limiting treatment options and complicating management strategies.

Similarly, the high prevalence of MDR uropathogens, especially *P. aeruginosa*, is a major concern in clinical microbiology, as highlighted by our study, which found a 26.5% MDR rate in this pathogen. This finding is consistent with existing literature highlighting significant MDR rates in various geographical contexts. For example, a large-scale multicenter study in Spain reported 26% of *P. aeruginosa* isolates as MDR, while a study in Italian teaching hospitals found a MDR rate of 26.9% [41, 42]. Conversely, the overall MDR rate across Europe is much lower (13.7%) [43].

The emergence of MDR strains is often linked to factors such as inappropriate antibiotic use and poor patient compliance, which exacerbate the problem of resistance in community settings. This issue is particularly relevant in Albania, where a study reported that 53.4% of the population used antibiotics without a prescription, a practice reportedly supported by Albanian pharmacists. Furthermore, a significant portion of individuals failed to complete their prescribed antibiotic courses, thereby contributing to resistance [44]. Similar concerns have been raised in hospital settings, where factors such as excessive empirical antibiotic use and transmission via medical devices have been reported as contributors to MDR prevalence of *P. aeruginosa* [45].

In summary, our study highlights the high prevalence of CA-UTIs in Albania, particularly among females, and underscores the importance of recognizing the diversity of uropathogens and their resistance patterns.

To improve the management of CA-UTIs and effectively address antibiotic resistance in Albania, several measures are recommended. Firstly, the development and implementation of standardized clinical protocols for diagnosing and treating CA-UTIs are essential. These protocols should be evidence-based and tailored to Albania's local epidemiological context, ensuring consistency in care and improving outcomes, particularly considering the high prevalence of MDR pathogens. Secondly, training programs for healthcare professionals should be prioritized to enhance their understanding of antibiotic stewardship and promote appropriate antibiotic prescription in treating UTIs. Such programs can address antibiotic misuse and resistance while fostering responsible prescribing practices. Finally, public awareness campaigns are necessary to educate the population on the risks of self-medication and the importance of adhering to prescribed treatments. Additionally, further multicenter and longitudinal studies are essential to improve generalizability and capture broader epidemiological trends across the country. These recommendations aim to strengthen Albania's healthcare response to CA-UTIs and mitigate the growing challenge of antibiotic resistance.

### 4.1. Strengths and Limitations

The present study addresses a critical research gap by examining the prevalence, resistance patterns, and MDR rate of nonenteric pathogens in CA-UTIs in Albania, a region with limited epidemiological data. The findings offer essential insights into the local epidemiology of UTI pathogens, contributing to evidence-based management strategies and public health policy development. The use of an ISO-certified laboratory, standardized culture methods, and the VITEK 2 automated system ensures high reliability and reproducibility of bacterial identification and susceptibility testing.

However, the study's single-center design may limit the applicability of findings to other regions or healthcare settings in Albania. Additionally, it does not account for socioeconomic or behavioral factors that could influence antibiotic resistance patterns. The cross-sectional nature of the study restricts its ability to capture temporal trends or seasonal variations in UTI prevalence and resistance.

## 5. Conclusions

In conclusion, this study underscores the high prevalence of CA-UTIs in Albania, particularly among females and younger individuals (18–39 years), highlighting the critical need for heightened awareness of the diverse uropathogens involved and their antibiotic resistance patterns. The significant identification of nonenteric pathogens coupled with the worrying rates of MDR strains, particularly in *P. aeruginosa,* which shows high resistance to fluoroquinolones and tigecycline, reflects the urgent challenges posed by antibiotic resistance in clinical practice. These findings stress the importance of establishing standardized protocols for diagnosis and treatment, enhancing antibiotic stewardship through targeted training for healthcare professionals, and fostering public awareness to combat self-medication practices. Moreover, further research is necessary to monitor evolving resistance patterns and ensure effective management of UTIs in the Albanian context. By implementing these recommendations, the healthcare system can enhance its response to CA-UTIs and mitigate the escalating threat of antibiotic resistance, ultimately improving patient outcomes and public health.

## Figures and Tables

**Table 1:** Distribution of microorganisms according to age and gender.

Pathogen type	Gender		Age group			Total *n* (%)
	Female *n* (%)	Male *n* (%)	18–39 years *n* (%)	40–64 years *n* (%)	65 or older *n* (%)	
*Enterococcus faecalis*	38 (67.9)	18 (32.1)	39 (69.6)	11 (19.6)	6 (10.8)	56 (54.4)
*Pseudomonas aeruginosa*	19 (55.9)	15 (44.1)	13 (38.2)	12 (35.3)	9 (26.5)	34 (33.0)
*Staphylococcus saprophyticus*	10 (83.3)	2 (16.7)	9 (75.0)	3 (25.0)	0 (0.0)	12 (11.6)
*Burkholderia cepacia*	1 (100.0)	0 (0.0)	1 (100.0)	0 (0.0)	0 (0.0)	1 (1.0)
Total *n* (%)	68 (66.0)	35 (34.0)	62 (60.2)	26 (25.2)	15 (14.6)	103 (100.0)

**Table 2:** Prevalence of antibiotic-resistant microorganisms stratified by pathogen type, antibiotic class, and demographics.

Pathogen type	Resistance rate	LVX	LZD	FEP	VAN	MEM	DAP	TG	TIM	PIP	TEC	TET
*E. faecalis*	Resistant (%)	14.3	17.9	—^1^	53.6	—	0.0	1.8	—	—	46.4	44.6
	Gender *p* value	**0.002**	3.118	—	0.098	—	NA	1.308	—	—	0.064	9.744
	Age group *p* value	2.299	2.199	—	1.185	—	NA	1.308	—	—	3.255	0.96

*P. aeruginosa*	Resistant (%)	38.2	—	2.9	—	0.0	—	91.2	26.5	26.5	—	—
	Gender *p* value	3.304	—	1.373	—	NA	—	3.387	**0.012**	**0.049**	—	—
	Age group *p* value	1.822	—	1.544	—	NA	—	1.599	2.302	1.305	—	—

*S. saprophyticus*	Resistant (%)	16.7	16.7	—	16.7	—	0.0	16.7	—	—	25.0	16.7
	Gender *p* value	**0.002**	3.118	—	0.098	—	NA	1.308	—	—	0.064	9.744
	Age group *p* value	2.299	2.199	—	1.185	—	NA	1.308	—	—	3.255	0.96

*B. cepacia*	Resistant (%)	0.0	—	0.0	—	0.0	—	0.0	0.0	0.0	—	—
	Gender *p* value	3.304	—	1.373	—	NA	—	3.387	**0.012**	**0.049**	—	—
	Age group *p* value	1.822	—	1.544	—	NA	—	1.599	2.302	1.305	—	—

*Note:* TIM: ticarcillin/clavulanic acid, PIP: piperacillin, FEP: cefepime, MEM: meropenem, LVX: levofloxacin, TG: tigecycline, LZD: linezolid, DAP: daptomycin, TEC: teicoplanin, VAN: vancomycin, and TET: tetracycline. The bold values in Table 2 refer to the *p*-values that are statistically significant.

Abbreviation: NA, not applicable.

^1^(—) Indicates either absence or a negative result.

## Data Availability

Research data are not shared.
